# Clustering of Expression Data in Chronic Lymphocytic Leukemia Reveals New Molecular Subdivisions

**DOI:** 10.1371/journal.pone.0137132

**Published:** 2015-09-10

**Authors:** Sally Yepes, Maria Mercedes Torres, Rafael E. Andrade

**Affiliations:** 1 Facultad de Ciencias, Departamento de Ciencias Biológicas, Universidad de los Andes, Bogotá D.C., Colombia; 2 Facultad de Medicina, Universidad de los Andes, Departamento de Patología, Hospital Universitario, Fundación Santa Fe de Bogotá, Bogotá D.C., Colombia; Queen's University Belfast, UNITED KINGDOM

## Abstract

Although the identification of inherent structure in chronic lymphocytic leukemia (CLL) gene expression data using class discovery approaches has not been extensively explored, the natural clustering of patient samples can reveal molecular subdivisions that have biological and clinical implications. To explore this, we preprocessed raw gene expression data from two published studies, combined the data to increase the statistical power, and performed unsupervised clustering analysis. The clustering analysis was replicated in 4 independent cohorts. To assess the biological significance of the resultant clusters, we evaluated their prognostic value and identified cluster-specific markers. The clustering analysis revealed two robust and stable subgroups of CLL patients in the pooled dataset. The subgroups were confirmed by different methodological approaches (non-negative matrix factorization NMF clustering and hierarchical clustering) and validated in different cohorts. The subdivisions were related with differential clinical outcomes and markers associated with the microenvironment and the MAPK and BCR signaling pathways. It was also found that the cluster markers were independent of the immunoglobulin heavy chain variable (IGVH) genes mutational status. These findings suggest that the microenvironment can influence the clinical behavior of CLL, contributing to prognostic differences. The workflow followed here provides a new perspective on differences in prognosis and highlights new markers that should be explored in this context.

## Introduction

Chronic lymphocytic leukemia (CLL) is one the most frequently occurring leukemias in adults in Western countries and is characterized by mature B cell accumulation in the blood, bone marrow and secondary lymphoid organs. CLL patients can be divided into two major groups based on whether their immunoglobulin heavy chain variable region (IGVH) genes are mutated or unmutated. Patients with an unmutated IGVH gene have a less favorable prognosis than patients with a mutated IGVH gene [1, 2]. Different chromosomal aberrations, such as deletions in 11q, 13q, or 17p and trisomy 12, have also been found in CLL patients, with varied prognostic implications [3]. Common genetic causes have not yet been identified [4], but recurrent mutations in *TP53* and *ATM* and new mutations in *NOTCH1*, *SF3B1*, *MYD88*, *BIRC3* and *FBXW7* have been identified in recent years by next-generation sequencing [5].

Little research has been performed to examine the natural clustering of CLL patient samples or to identify subtypes based on gene expression patterns, partly because expression studies in CLL patients have focused on the analysis and comparison of established disease subtypes. However, the identification of CLL patient groups is a current research goal, the realization of which could contribute to the identification of different prognostic subtypes and help to explain the heterogeneity in the clinical behavior of the disease. The main purpose of this study was to assess the possibility of detecting molecular subtypes of CLL patients based on gene expression microarrays in a relatively large group of samples obtained by merging expression readouts. If so, the goal was to confirm subdivisions in different cohorts, identify markers in the detected subgroups and explore the clinical and biological implications.

**Fig 1:**
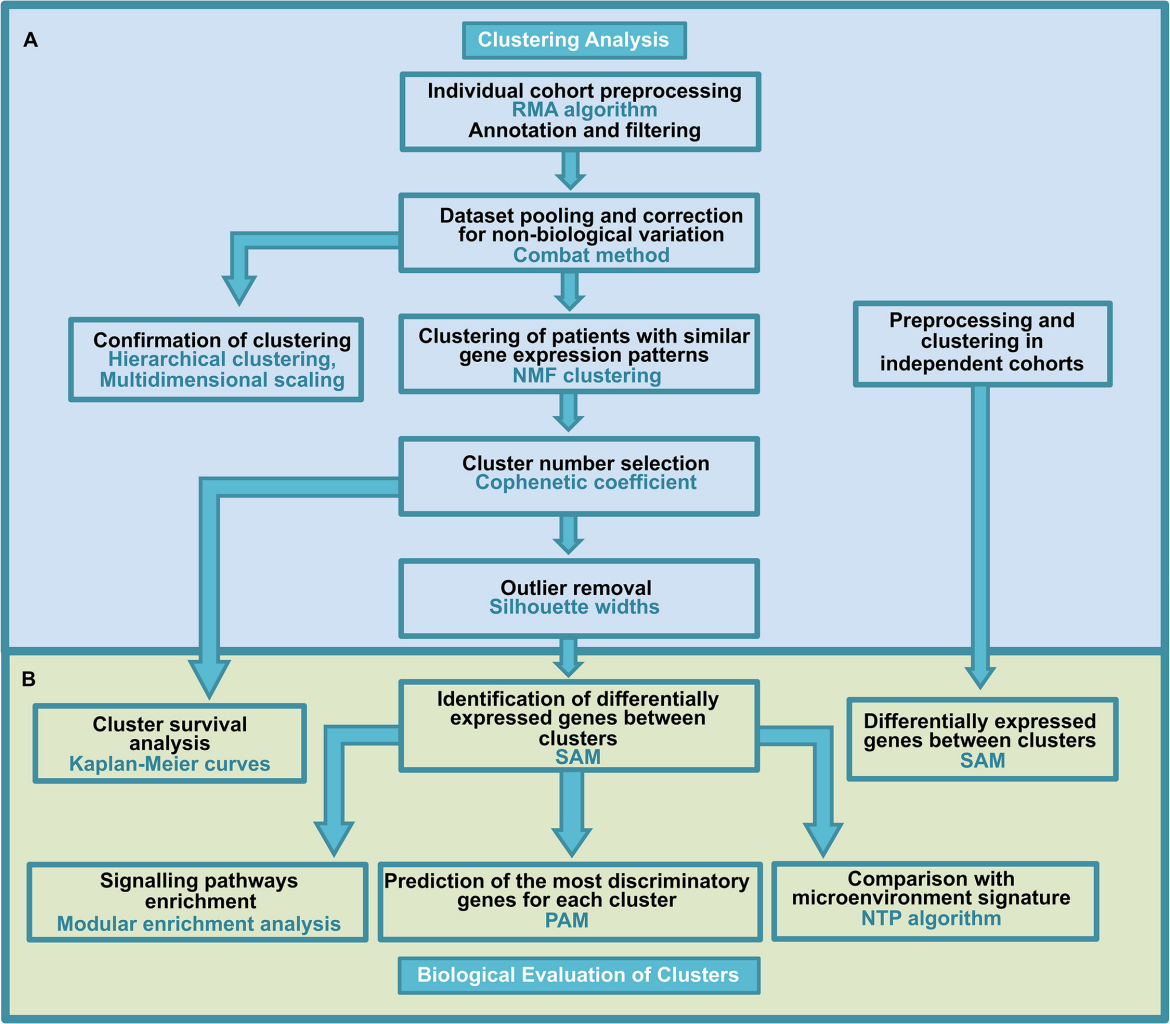
Methodology flow chart. Panel A shows the methodological steps followed for clustering analysis, and panel B shows the steps for the biological evaluation of the clusters obtained.

We followed the methodological workflow presented in Fig 1. Briefly, microarray datasets from two different CLL expression studies were individually preprocessed, merged and corrected for non-biological variation. The resulting pooled data were used to identify stable clusters, or subgroups of patients with similar gene expression patterns. To this end, we applied different unsupervised clustering methods to confirm the structure in the data (non-negative matrix factorization NMF clustering, hierarchical clustering and multidimensional scaling). Cluster analysis was performed in 4 other independent cohorts. To identify cluster-specific genes, we identified genes that were differentially expressed between the clusters using the significance analysis of the microarray SAM method in both the merged data and individual cohorts.

The resulting genes were analyzed in relation with the biology of the disease, pathway enrichment, and predictive role. The survival implication of the clusters and the individual contribution of cluster-specific markers to survival were evaluated. The relationship of the clusters to IGVH mutational status was also analyzed. A detailed explanation of the methodology can be found in the Materials and Methods section.

## Materials and Methods

A schematic description of the workflow is presented in Fig 1. Panel A shows the methodological steps followed for clustering analysis, and panel B shows the steps for the biological evaluation of the clusters.

### Clustering Analysis

#### Dataset and Array Preprocessing

The present study used microarray expression profiles of CLL obtained from the Gene Expression Omnibus (GEO) database of the National Center for Biotechnology Information (NCBI). GSE39671: (n = 130) [6] and GSE22762: (n = 107) [7] (both analyzed with the Affymetrix Human Genome U133 Plus 2.0 Array) were chosen for clustering analysis because they contain data about time to treatment (TTT) and overall survival (OS), respectively, and have suitable, comparable and large numbers of samples. They were independently preprocessed before combining them in one dataset for clustering and subsequent analysis.

Another 4 cohorts that were analyzed using different microarray platforms were also chosen and preprocessed for clustering analysis and to explore the relationship with IGVH mutational status. These cohorts correspond to the following: GSE46261: (n = 211), Affymetrix Human Gene 1.0 ST Array [8], GSE9992: (n = 60), Affymetrix Human Genome U133A Array [9], GSE2466: (n = 100), Affymetrix Human Genome U95A Array [10] and GSE38611: (n = 136) Affymetrix Human Gene 1.0 ST Array [11].

Once the studies were selected, raw gene expression data from each study were independently preprocessed; this process comprised 3 steps: 1) background correction to adjust the intensity readings for nonspecific signals; 2) adjustment of the intensity readings for technical variability to ensure that the measurements of all of the samples were comparable (normalization); and 3) computation of a summary value for the different probes representing each gene (summarization). Each probe was also linked to its corresponding gene name (annotation), and non-relevant information was removed (filtering) [12]. Individual cohort preprocessing was performed using the RMA algorithm, a method that encompasses all 3 preprocessing steps [13].

Entrez gene IDs for Affymetrix probes were obtained from the appropriate annotation package for each microarray platform. Gene filtering removed 10% of the unexpressed and non-informative genes [14]. All of the analyses were performed using the appropriate package in R [15]. Specifically, the ‘affy’ package was used for microarray reading and for the initial preprocessing steps [16]. Gene annotation was performed using the annotation package [17]. Quality control was performed with affyQCReport [18], and the filtering procedure was performed with MetaDE software [19].

#### Dataset Pooling

Combining data from different studies can be beneficial for uncovering underlying biological insights that are not easily identified in few cases and can increase the statistical power of the study. However, because non-biological experimental variation or “batch effects” are observed across independent experiments, after merging cohorts, it is necessary to correct for systematic variation without compromising the structure of the data or the biological information contained within the data. Here, the cohorts GSE39671 and GSE22762 were merged and corrected for non-biological variation using The COMBAT method (empirical Bayes) implemented in the inSilicoMerging package [20].

#### Consensus-based Non-negative Matrix Factorization (NMF)

To predict stable clusters in the merged data, NMF was applied, which detects context-dependent patterns in gene expression data rather than dividing clusters based on distance computation. NMF is based on the decomposition of data into parts and can reduce the dimensionality of an expression set from thousands of genes to several metagenes. Each metagene is defined as a positive linear combination of genes in the expression data. NMF then groups the samples into clusters based on the gene expression pattern of the samples as positive linear combinations of these metagenes. NMF Consensus repeatedly runs the clustering algorithm against perturbations of the gene expression data and creates a consensus matrix to assess the stability of the resulting clusters [21].

Let *X* be an nxp non-negative matrix and r>0 be an integer. Non-negative matrix factorization consists of finding an approximation

X≈WH,

where *W* and *H* are nxp and rxp non-negative matrices, respectively. Because the objective is to reduce the dimensionality of the original data, the factorization rank r is often used, such that r << min(n, p). The objective behind this choice is to summarize and split the information contained in *X* into r factors: the columns of *W*. The main approach to NMF is to estimate the matrices *W* and *H* as a local minimum:

$$\begin{array}{c} \underset{W,H\geq 0}{\min}\;\left[D\left(X,W\;H\right)+R\left(W,H\right)\right],\\ \;=F\left(W,H\right) \end{array}$$

where *D* is a loss function that measures the quality of the approximation. Common loss functions are based on the Frobenius norm or the Kullback-Leibler divergence. The NMF algorithm was applied in GenePattern software [22].

#### Cluster Number Selection and Outlier Removal

Selection of the number of classes or clusters was performed using the quantitative Cophenetic coefficient defined in Brunet et al [21]. The Cophenetic coefficient computes a score of global clustering robustness across the consensus matrix. The number of clusters was also confirmed for inspection of the graphical representation of the consensus matrix.

Even though clustering methodologies using the consensus process can detect robust groups, the identification of cluster-associated genes can be influenced by unusual samples. To minimize the impact of outliers on cluster marker identification, samples with negative silhouette widths were excluded, and only samples that were significantly associated with the center of each cluster were included; this was performed using the cluster package [23].

#### Hierarchical Clustering and Multidimensional Scaling

To corroborate the subgroup structure in the data, in addition to the NMF method, we also applied different methodological approaches such as hierarchical clustering and multidimensional scaling. Preprocessed expression arrays were subjected to hierarchical clustering using the Ward method and the distance 1-r, where r is the Pearson correlation coefficient. Multidimensional scaling was applied to visualize subdivisions in the merged data and to evaluate the distance used for the hierarchical clustering. The analysis was performed using the cluster package [23].

### Biological Evaluation of Clusters

#### Cluster Markers

To identify cluster-specific genes, we identified genes that were differentially expressed between clusters using significance analysis of microarray (SAM) [24], allowing the identification of up-regulated and down-regulated genes in each cluster. This method assesses differential gene expression relative to the spread of expression across all genes. The false discovery rate (FDR) was set to 0. The analysis was performed using the siggenes package [25].

The markers obtained from SAM, using the merged data, were analyzed and used to predict the more discriminatory genes for each cluster. To accomplish this, we used Prediction Analysis for Microarrays (PAM), in which the nearest shrunken centroid for the data was computed [26]. Leave one out cross validation (LOCV) was applied to cross-validate the classifier produced. The procedures were executed in the pamr package [27].

#### Functional Enrichment

To identify signaling pathways involved in the differences between clusters, the differentially expressed genes identified with SAM were analyzed for modular enrichment using the Genecodis server [28, 29, 30]. The method obtains co-occurrence annotations in the KEGG and Panther databases, the P values are calculated through hypergeometric analysis corrected by FDR method

#### Nearest Template Prediction (NTP) and Microenvironment Signature

To associate the class of a given sample (cluster membership) to a CLL microenvironment signature, the nearest template prediction algorithm (NTP) [31] was applied to the merged dataset using GenePattern software [22]. To obtain CLL microenvironment signatures, the original microarray data from Herishanu et al [32] were used. Matched tissue and blood samples that were simultaneously obtained from CLL patients were preprocessed and analyzed to identify genes that were differentially expressed between the lymph nodes (LN) and peripheral blood (PB), and genes that were differentially expressed between bone marrow (BM) and PB. Differential expression was assessed by SAM analysis (>2-fold change, FDR <20%). A B-cell receptor (BCR) signature obtained by Pede et al [33] was used after BCR stimulation of CLL cells for 24 hours, and genes with a fold change >2 were considered.

#### Survival Analysis

Patients from cohorts GSE39671 and GSE22762 were used to determine whether the obtained clusters were related to survival (TTT and OS). Survival curves were analyzed according to the Kaplan-Meier method and compared using the log-rank test. To evaluate the contribution of individual genes to survival, Cox regressions were applied. The analyses were performed using the survival package [34]. Given the relevance of the IGVH mutational status for the prognosis of CLL, the relationship between clusters and the mutational status was evaluated in the 4 independent cohorts.

#### Heatmaps

Heatmaps were generated with Gene Pattern software. The genes in the heatmaps were ordered based on their differential expression using a t test [22].

## Results

### Primary Cluster Identification in CLL

The use of small cohorts can prevent the identification of subgroups that are revealed when a large and heterogeneous group of samples is employed. Therefore, in this paper, we combined information from different and independent expression cohorts to increase the statistical power of the study. We independently preprocessed the expression datasets GSE22762 and GSE39671; both of these cohorts were originally assayed using the same microarray platform (Affymetrix Human Genome U133 Plus 2.0 Array). After preprocessing, we obtained 16,287 genes for each dataset. We merged the above studies and adjusted for non-biological variation, obtaining a list of 15,895 genes in common between the two studies and 237 samples in total. This data matrix is available as **S1 Table.**

**Fig 2:**
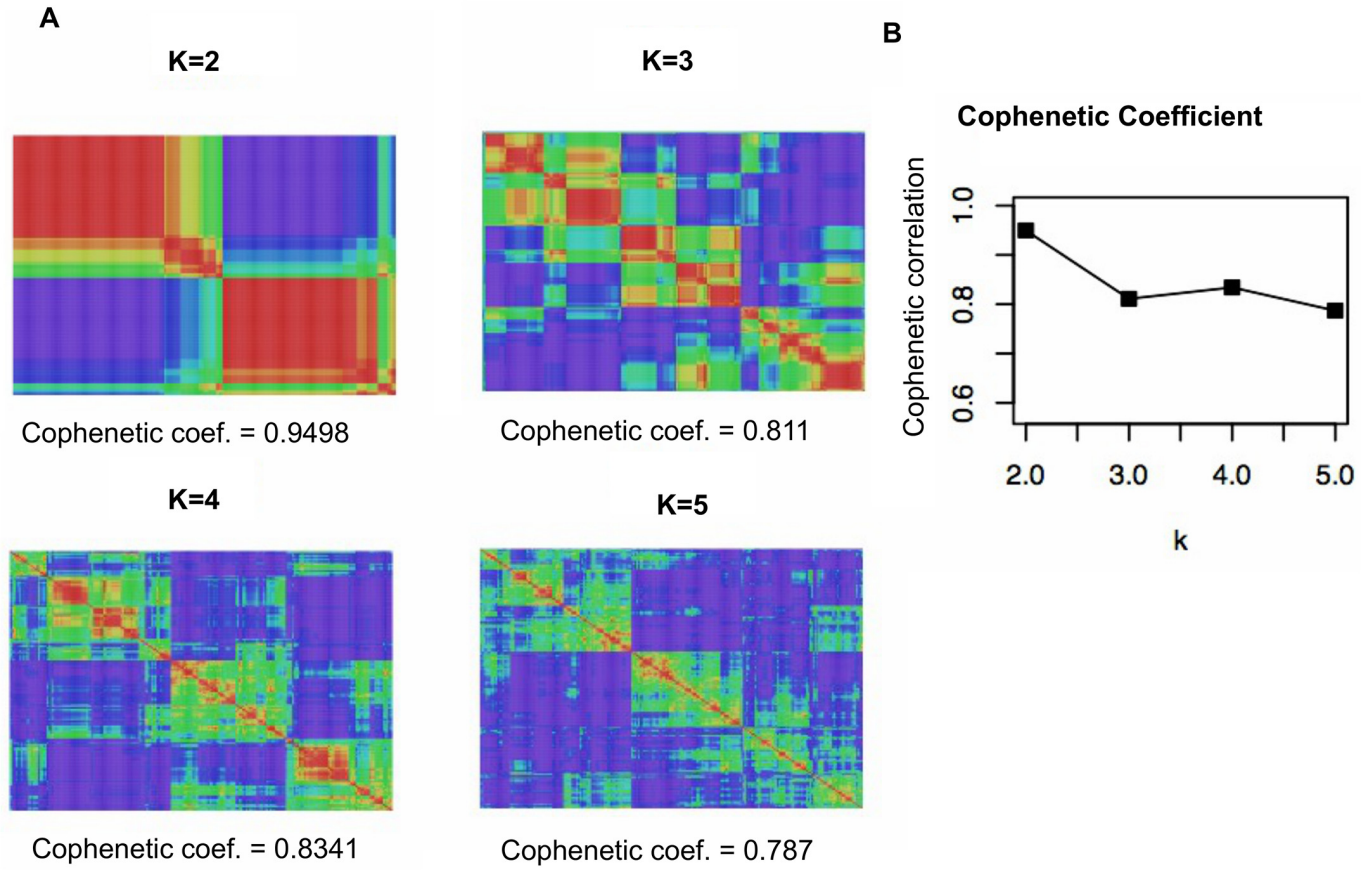
NMF consensus clusters for the pooled data. NMF consensus analysis of the merged data revealed a good consensus for k = 2. A. Maximum cophenetic coefficients for k = 2 to 5 clusters and the consensus matrices for k = 2 to 5 are shown. B. Plot showing a comparison of cophenetic coefficients among k clusters. This score provides a summary of global clustering robustness across the consensus matrix, with zero indicating the least robust partition and one indicating the most robust partition. From the perspective of robustness, the maximum peak of the cophenetic coefficient plot determines the optimal number of subgroups in the data. The division in the data is also evident in the consensus matrix, which showed a clear boundary between red and blue areas, indicating robust and stable clustering in comparison with other subdivisions.

We used the NMF clustering method to cluster the described merged data according to gene expression and identify patient subtypes. The NMF analysis defined two distinct high-consensus CLL subgroups (Fig 2), to which we refer as cluster 1 and cluster 2. The subdivision is evident when visualizing the consensus matrix (Fig 2A) and based on the highest value of the global clustering robustness score for k = 2 (Fig 2B). Detailed clustering analysis results are presented in **S2 Table** and graphically represented in **S1 Fig**, in which the identity of the samples, the cohort from which they were derived and their class membership after clustering with NMF are shown.

**Fig 3:**
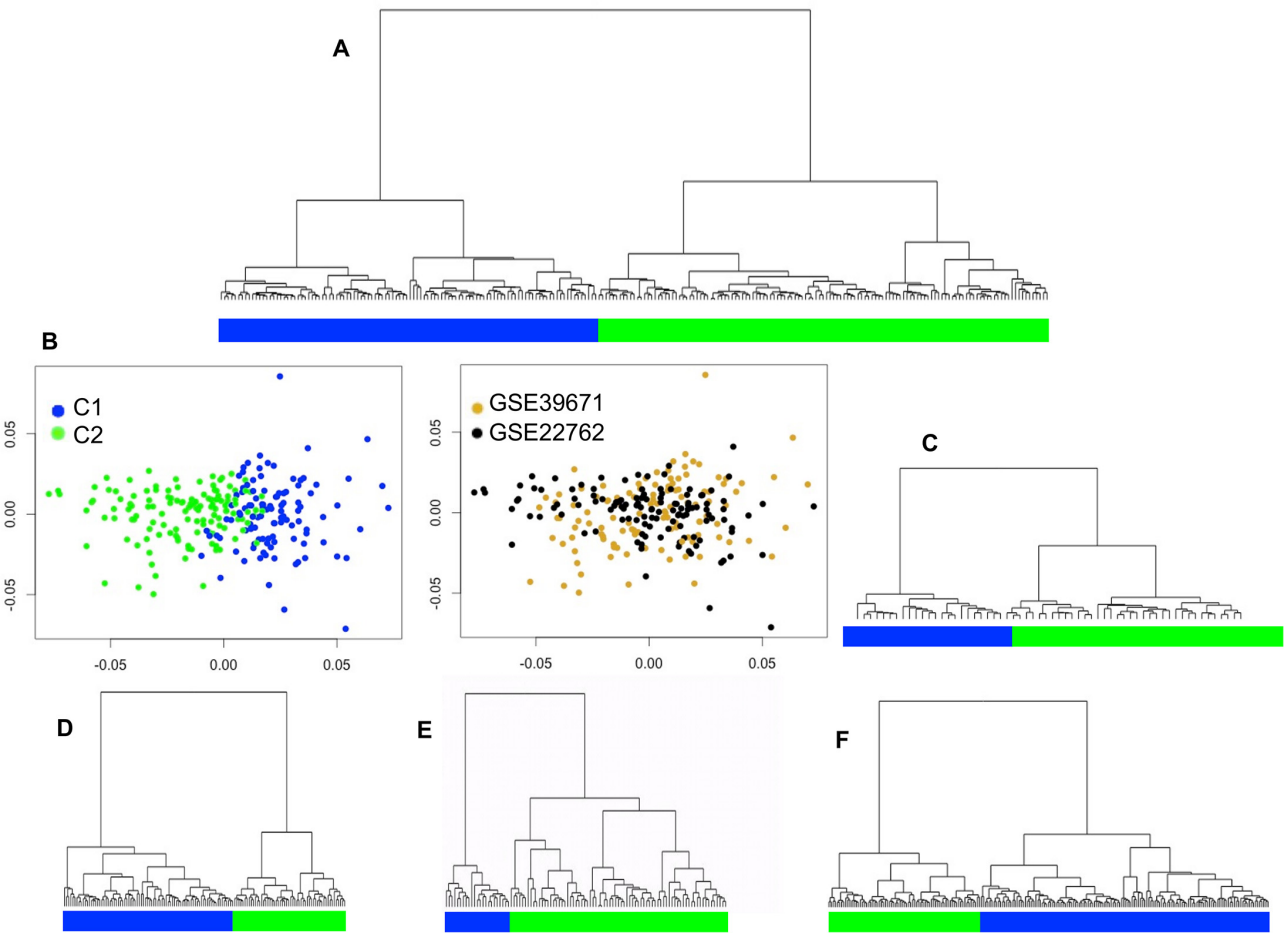
CLL sample clustering showing the primary transcriptional subgroups. Dendrogram obtained by hierarchical clustering of different cohorts. A. Merged dataset. B. Multidimensional scaling of the merged data; the left panel shows evidence of two clusters, and the right panel shows the two individual cohorts after sample merging. However, some sample overlap between groups was evident. C. Cohort GSE38611. D. Cohort GSE9992. E. Cohort GSE2466. F. Cohort GSE46261.

To corroborate the partition of the samples into two different subtypes, we applied hierarchical clustering. We found class membership coincidence between the NMF consensus clustering and the hierarchical clustering in 90% of the samples, as most samples belonged to the same clusters in both analyses. This result supports two major subdivisions in the data. Furthermore, hierarchical clustering allowed the two subgroups in the 4 independent cohorts to be individually analyzed (Fig 3A and 3C–3F).

Multidimensional scaling was useful for evaluating the adequacy of the distance used in the hierarchical clustering and made it possible to visualize two clusters in the data. This analysis also revealed a lack of sample grouping on the basis of sample origin, confirming that the pooled samples had been properly adjusted for batch effects (Fig 3B).

In conclusion, the merged expression dataset that represents a large and heterogeneous group of patients with CLL can be naturally divided into two stable and robust transcriptional subgroups. This subdivision is supported by methodological approaches of a different nature (NMF clustering detects context-dependent patterns and hierarchical clustering divides data based on distance computation) and was also confirmed in 4 independent cohorts.

### Cluster-specific Marker Identification

We identified markers associated with the CLL subtypes by searching for genes that were differentially expressed (DE) between the two clusters. The SAM analysis identified up to 3379 genes with a statistically significant difference between clusters in the merged dataset (ΔSAM = 4 and FDR = zero). Under a more stringent cut-off (corrected P value = 0 and median fold change >2), which may reflect a more biologically relevant scenario, we identified 230 genes that were differentially expressed between clusters.

Some of the most highly up-regulated genes in cluster 2 were *FCRLA*, *HDHD2*, *TCL1A*, *TNFRSF17* and *SERPINI1*; conversely, these genes were down-regulated in cluster 1. The most up-regulated genes in cluster 1 include *SERPINB2*, *DENND4B*, *C15ORF48*, *ZNF331* and *NR4A2*; conversely, their expression was down-regulated in cluster 2. The most highly up-regulated genes in each cluster and their fold changes are presented in Table 1. A complete list of all differentially expressed genes in each cluster as well as their statistical parameters and fold changes can be found in the supplementary information (**S1 File**).

**Table 1:** Most highly up-regulated genes in each cluster (merged data).

	Cluster 2		Cluster 1	
	Gene	Fold change	Gene	Fold change
1	*FCRLA*	4,51	*SERPINB2*	5,93
2	*HDHD2*	4,45	*DENND4B*	5,53
3	***TCL1A***	4,15	*C15ORF48*	5,36
4	*TNFRSF17*	3,93	*ZNF331*	5,20
5	*SERPINI1*	3,51	*NR4A2*	4,31
6	*ANXA4*	3,28	*G0S2*	4,28
7	*UGDH*	3,18	*METRNL*	3,91
8	*GAPT*	3,16	*SLC7A5*	3,60
9	*AIM2*	3,15	*MAFB*	3,43
10	*CPNE5*	3,10	*IL1B*	3,42

**Fig 4:**
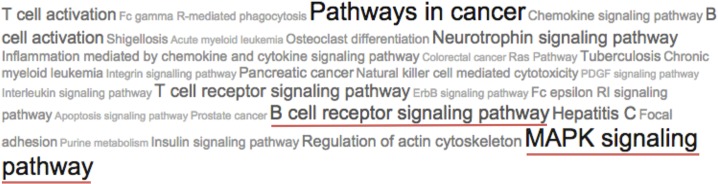
Tag cloud representing the most significant terms enriched in the list of DE genes. The sizes of the tags vary according to the number of supporting genes found in each enriched term.

Importantly, the most significantly differentially expressed genes between clusters, obtained either from the merged data or individual cohorts, showed outstanding reproducibility (Table 2). The common genes found to be up-regulated in cluster 2 and conversely down-regulated in cluster 1 include *TCL1A*, *FCRLA*, *FIG4*, *AIM2*, *SELL*, *RAC2*, *CD27*, *SAMD9L*.

**Table 2:** Most highly up-regulated genes in cluster 2 (independent cohorts).

	GSE39671	GSE22762	GSE9992	GSE46261	GSE24666	GSE38611
1	*TNFRSF17*	***FCRLA***	***TCL1A***	*FCRL1*	*METTL7A*	***FCRLA***
2	***TCL1A***	***TCL1A***	*SELL*	*FCRL5*	***TCL1A***	*SAMD9L*
3	***FCRLA***	*HDHD2*	*TGFBI*	***FCRLA***	*CD79B*	*FCRL1*
4	*HDHD2*	*SERPINI1*	*AIM2*	*PDGFD*	*PTPN6*	*FCRL5*
5	*DYNLL1*	*ANXA4*	*CD79B*	*ZMAT1*	*SYK*	***TCL1A***
6	*CDC20*	*HIBCH*	*FAM65B*	*NDRG3*	*SKAP2*	*SLAMF6*
7	*IRF2*	*SLC25A43*	*TRAC*	*SAMD9L*	*CD27*	*FIG4*
8	*ZNF559*	*FIG4*	*C17ORF62*	*FCRL2*	*RAC2*	*FCRL2*
9	*HIST1H2AC*	*C17ORF62*	*P2RY14*	*NIPAL2*	*FAM65B*	*NDRG3*
10	*UGDH*	*CPNE5*	*PSMB9*	*CCDC141*	*ACADM*	*LYST*

The proto-oncogene *TCL1* is of particular interest due to its crucial role in CLL pathogenesis. A high level of expression of this gene is associated with CLL development [35, 36, 37]. Recently, it has been demonstrated that stromal cells modulate *TCL1* expression in CLL and repress important target molecules such as *FOS*, *JUN* and members of the AP-1 complex, suggesting that microenvironment-derived signals play an important role in the survival of CLL cells [38]. *TNFRSF17* was the first up-regulated gene identified in experiments in which CLL cells were co-cultured with different stromal cells [38]. This gene was also identified as one of the most significantly differentially expressed genes between clusters in the merged data, supporting the influence of stromal cells on cluster 2.

FCRL family of proteins showed differentially expression between clusters, these proteins share many similar features with the classical Fc receptors and some members of this family have predictive value for determining clinical progression in CLL [39]

Given the highly differential expression of *TCL1* between clusters, its repeatable expression pattern in different cohorts, and its role in the microenvironment and CLL progression, we call attention to the biological implication of this gene in cluster subdivision.

Given the large number of genes that are differentially expressed between clusters and for the purpose of proposing reliable cluster markers, we employed a prediction method (PAM) to find the most discriminatory genes. From the 230 genes that were differentially expressed between clusters, the method could identify a minimal set of 34 genes capable of predicting, with an overall error rate of less than 5%, the cluster membership. The resulting markers ordered by PAM score and showing the direction of gene expression are listed in Table 3. Based on our analyses, the highest score was assigned to *ZNF331* as a predictive marker of clusters 1. *ARID5A*, *C15ORF48*, *SLC7A5*, *ELL2*, *MTMR6*, were also assigned to this cluster. *HDHD2*, *UGDH*, *TNFRSF17*, *FCRLA C11ORF73*, *ZNF559*, and *TCL1A*, among other genes, were assigned to cluster 2. Interestingly, one of the most biologically relevant genes in the cluster 2, *TCL1A*, has roles as proto-oncogene, and the gene with the highest discrimination score in the cluster 1, *ZNF331*, has roles as tumor suppressor gene.

**Table 3:** Cluster-specific markers after prediction analysis-PAM.

	Gene	PAM score for cluster 2	PAM score for cluster 1	Fold change cluster 2 vs. 1	Fold change cluster 1 vs. 2
1	*ZNF331*	-0,1901	0,1527	0,19	5,20
2	*HDHD2*	0,1616	-0,1298	4,45	0,22
3	*UGDH*	0,0869	-0,0698	3,18	0,31
4	*TNFRSF17*	0,0850	-0,0682	3,93	0,25
5	*FCRLA*	0,0762	-0,0612	4,51	0,22
6	*C11ORF73*	0,0709	-0,0569	2,72	0,37
7	*ZNF559*	0,0496	-0,0398	3,01	0,33
8	*ARID5A*	-0,0495	0,0398	0,36	2,81
9	*TCL1A*	0,0414	-0,0332	4,15	0,24
10	*SERPINI1*	0,0413	-0,0332	3,51	0,28
11	*RP11-35G9*.*3*	0,0404	-0,0325	2,84	0,35
12	*MSH2*	0,0384	-0,0309	2,86	0,35
13	*ACADM*	0,0379	-0,0305	3,01	0,33
14	*FIG4*	0,0363	-0,0291	2,90	0,34
15	*C15ORF48*	-0,0294	0,0236	0,19	5,36
16	*HIBCH*	0,0228	-0,0183	3,03	0,33
17	*SLC7A5*	-0,0224	0,0180	0,28	3,60
18	*C17ORF62*	0,0216	-0,0173	2,87	0,35
19	*RNASEH2A*	0,0173	-0,0139	2,41	0,42
20	*ELL2*	-0,0166	0,0134	0,38	2,60
21	*ATG4C*	0,0141	-0,0114	3,02	0,33
22	*SAMD9L*	0,0128	-0,0103	2,91	0,34
23	*GOLPH3L*	0,0104	-0,0084	2,62	0,38
24	*STX7*	0,0094	-0,0076	2,59	0,39
25	*MTMR6*	-0,0089	0,0072	0,39	2,57
26	*HDDC3*	0,0083	-0,0067	2,35	0,43
27	*ZDHHC16*	0,0077	-0,0062	2,58	0,39
28	*TBCK*	0,0062	-0,0050	2,73	0,37
29	*CYB561A3*	0,0060	-0,0049	2,45	0,41
30	*CHD1*	-0,0051	0,0041	0,44	2,28
31	*AIM2*	0,0044	-0,0035	3,15	0,32
32	*ACOT13*	0,0023	-0,0018	2,45	0,41
33	*ACYP1*	0,0011	-0,0009	2,76	0,36
34	*FCGR2B*	0,0011	-0,0008	2,88	0,35

Fold change from SAM

We conclude that the similarity in different cohorts with regard to differential expression patterns reflects the robustness in the group structure (i.e., the presence of two subtypes of patients), and we suggest that important genes such as *TCL1A* and *ZNF331* are accountable for the biological subdivision.

#### Functional Enrichment

When analyzing the total list of genes that were differentially expressed between clusters through functional enrichment, many co-occurring annotations were found. The top annotations or terms, in order of corrected P values, were amino acid degradation (2.87361e -14), purine and pyrimidine metabolism (3.32583e-13 and 1.00552e-11, respectively), B cell receptor signaling pathway (6.55445e-11), protein processing in endoplasmic reticulum (8.21315e-11), RNA degradation (2.76037e-10), and RNA transport (6.45845e-10). MAPK signaling also had a significant P value (1.52119e-06).

Given the importance of signaling pathways in cancer, we enlisted the genes identified in the analysis that were involved in the BCR and MAPK signaling pathways. Genes involved in the BCR signaling pathway included *MAPK1*, *CR2*, *CD19*, *BTK*, *PIK3R5*, *SYK*, *NFKB1*, *VAV1*, *AKT1*, *CD79B*, *NFATC1*, *PPP3CB*, *PIK3CA*, *BLNK*, *FCGR2B*, *MAP2K2*, *PIK3R2*, *IKBKB*, *PIK3AP1*, *RELA*, *RAF1*, *NRAS*, *SOS1*, *NFKBIB*, *NFATC2*, *PIK3R1*, *RAC2*, *PTPN6*, *PPP3CA*, *PRKCB*, and *NFATC3*. Genes involved in MAPK signaling included *MAPK1*, *BRAF*, *MAPK9*, *PAK2*, *NFKB1*, *AKT1*, *PPP3CB*, *MAP2K2*, *IKBKB*, *RAF1*, *SOS1*, *NFATC2*, *NFKB2*, *CDC42*, and *PPP3CA*, among others. When the number of differentially expressed genes was reduced to include only those with the largest differences in expression, the above annotations were maintained with significant P vales, and the BCR and MAPK pathways are highlighted (Fig 4).

It is possible that differences in the clusters are due to the B-cell receptor (BCR) activation, which can trigger the activation of downstream signaling pathways such as the MAPK pathway. This response can vary depending on the cellular microenvironment.

#### Microenvironment Signature Activation

Because the tumor microenvironment may contribute to CLL pathogenesis, we searched for possible microenvironment associations in the data. To associate the samples with a CLL microenvironment signature, we used the NTP algorithm. We used the raw data from Herishanu et al [30] to obtain a microenvironment signature and found 86 differentially expressed genes between the LN and PB (>2-fold change, FDR <20%) (**S3 Table**). Many of the genes that were overexpressed in the LN are considered BCR target genes. Functional analysis of this microenvironment signature using Genecodis software identified a set of BCR-related genes as the most overrepresented; the NF-κB and NFAT pathways were also represented, both of which are activated by BCR signaling. Therefore, the microenvironment signature obtained here indicates the activation of distinct signaling pathways and tumor proliferation in the LN, as reported previously by Herishanu et al [32].

**Fig 5:**
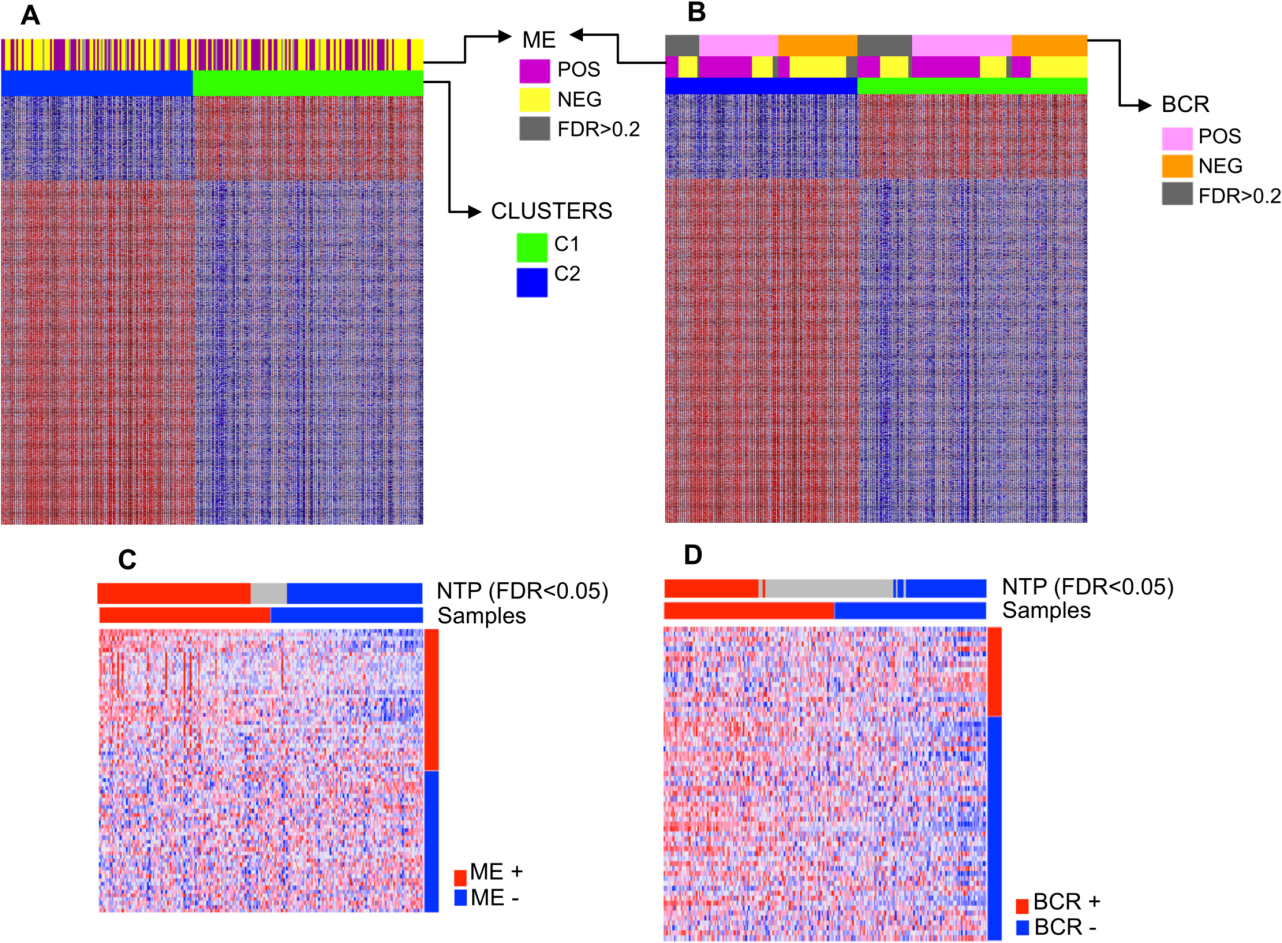
Heatmaps showing the association of clusters with A. microenvironment (ME) and B. BCR stimulation signatures. Both clusters showed a degree of ME and BCR signaling activation. Nearest template prediction (NTP) using C. microenvironment and D. BCR stimulation signature.

After NTP, 88.6% of the samples were assigned to one of two possible signature classes using FDR <0.05, and 93.4% of the samples were assigned to one of two possible signature classes using FDR <0.2. The CLL samples were enriched for the microenvironment-related signature, even though a relationship with specific clusters was not clearly found (Fig 5). It was possible to find agreement between the prediction made by the method (signature class assigned) and the two clusters in up to 56% of the cases. We also used the BCR stimulation signature previously described by Pede V et al [33] and observed a less confident prediction (80.3% of samples using FDR <0.2 and 57.6% of samples using FDR <0.5) and a lack of clear association with the clusters. The tested bone marrow signature also failed to show a clear relationship with a specific cluster.

The division of molecularly heterogeneous samples into two clusters can be influenced by multiple and complex processes, including the influence of the cell microenvironment. Additionally, signatures applied in the prediction method are very particular and specific. Therefore, it was not possible to link all the samples to the microenvironment signature tested.

### Clustering and Survival Analysis

**Fig 6:**
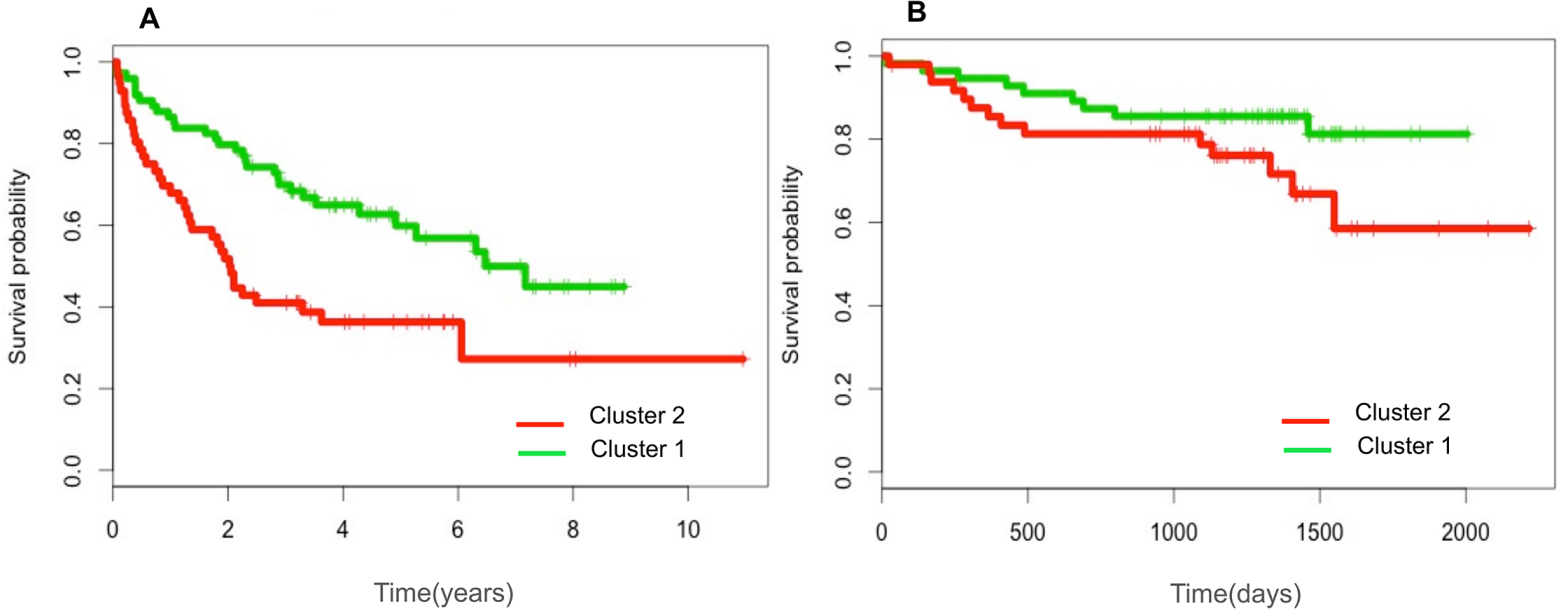
Clinical outcomes of the patients in the two cohorts. A. GSE39671, time to treatment (TTT) according to the cluster 1 and cluster 2. B. GSE22762, overall survival (OS) according to the cluster 1 and cluster 2.

To evaluate the clinical relevance of the clustering, we assessed cluster membership in relation to overall survival and time to treatment using the GSE22762 and GSE39671 datasets, respectively. Kaplan-Meier curves showed that the cluster 2 patients had poorer outcomes compared to the patients of cluster 1 (Fig 6). We compared the two groups using the log-rank test to evaluate the prognostic value of the model, and this analysis revealed a highly significant difference between expression levels and TTT and a nearly significant difference in OS.

To evaluate the contribution of individual genes to the prognostic difference between clusters, we applied Cox regressions to 230 genes (genes with the highest expression differences between clusters, P value = 0 and median fold change >2). The analysis confirmed the results of the Kaplan-Meier curves: the two clusters showed prognostic differences, and almost all of the up-regulated genes in each cluster have the same relationship with survival (i.e., negative for cluster 2 and positive for cluster 1) (**S2 File**).

Genes with statistically significant differences for both survival indicators (TTT and OS) can be considered highly informative of survival and are listed in Table 4. *NRIP1* and *MAFB* from cluster 1 are highlighted due to their lower P values and positive relationship.

Of the 230 genes analyzed, it was found that several genes were associated with TTT and OS (111 and 101, respectively). A total of 83 genes had a negative relationship with TTT, and 28 genes had a positive relationship with TTT, whereas 60 genes had a negative relationship with OS and 41 had a positive relationship with OS (**S2 File**).

**Table 4:** Genes showing common statistically significant differences for TTT and OS.

	Cluster 2					Cluster 1			
	Gene	survival outcome	TTT	OS		Gen	survival outcome	TTT	OS
1	*FCRLA*	neg	4,79E-03	6,74E-04	1	*SERPINB2*	pos	5,90E-03	5,28E-04
5	*SERPINI1*	neg	2,41E-02	3,75E-02	2	*DENND4B*	pos	5,84E-03	2,39E-04
10	*CPNE5*	neg	1,53E-02	4,30E-03	3	*C15ORF48*	pos	4,87E-02	1,02E-02
11	*HIBCH*	neg	4,84E-03	1,29E-02	6	*G0S2*	pos	3,33E-03	5,01E-03
18	*FCGR2B*	neg	1,20E-03	1,02E-03	9	*MAFB*	pos	3,40E-03	4,49E-07
21	*RP1135G93*	neg	3,25E-02	9,26E-03	10	*IL1B*	pos	1,23E-02	1,16E-03
26	*NAPSB*	neg	1,69E-02	1,27E-03	13	*NINJ1*	pos	1,53E-03	5,10E-03
30	*CD27*	neg	2,64E-03	4,53E-04	14	*NRIP1*	pos	1,23E-02	1,89E-10
35	*DYNLL1*	neg	4,54E-03	1,09E-02	15	*PFKFB3*	pos	1,20E-02	1,96E-03
36	*ZDHHC16*	neg	2,49E-02	3,23E-02	18	*IER3*	pos	3,90E-03	2,03E-04
37	*SPIB*	neg	1,21E-02	8,54E-03	19	*SGK1*	pos	2,91E-02	6,59E-05
38	*TMEM14C*	neg	4,84E-03	3,69E-02	20	*THBS1*	pos	2,04E-03	2,14E-04
43	*TMEM251*	neg	4,63E-03	4,33E-02	23	*IL8*	pos	3,58E-03	1,94E-03
47	*ACOT13*	neg	1,53E-02	1,30E-02	25	*C5AR1*	pos	3,36E-03	2,75E-03
49	*RAC2*	neg	6,56E-03	9,14E-03	29	*GNA15*	pos	7,37E-03	1,06E-03
51	*RNASEH2A*	neg	4,50E-03	6,34E-03	34	*FOSL2*	pos	3,73E-02	4,59E-03
58	*HDDC3*	neg	1,35E-02	3,34E-02	35	*TREM1*	pos	1,59E-02	3,13E-02
59	*KIAA1407*	neg	9,41E-03	3,59E-03	40	*THBD*	pos	8,48E-03	6,87E-03
62	*AIDA*	neg	6,66E-04	4,71E-02	41	*UPP1*	pos	8,78E-03	1,12E-04
64	*VPREB3*	neg	2,16E-02	3,08E-03	43	*CCR1*	pos	1,53E-02	7,36E-04
78	*FCRLB*	neg	2,71E-03	3,52E-04	53	*PLAUR*	pos	4,95E-03	3,57E-03
79	*DAD1*	neg	4,63E-02	2,19E-02	56	*WHAMM*	pos	3,51E-02	7,29E-03
86	*RUVBL1*	neg	1,82E-03	8,55E-03	75	*SMIM3*	pos	1,11E-02	2,38E-04
93	*ECHS1*	neg	1,44E-02	9,43E-03					
111	*CDK2AP2*	neg	7,40E-03	3,81E-02					
116	*MPV17*	neg	2,42E-02	3,57E-04					
120	*RP5886K23*	neg	1,96E-02	2,20E-04					
121	*CISD1*	neg	2,44E-03	5,61E-03					
126	*ECI1*	neg	1,19E-02	8,14E-05					
138	*AP2B1*	neg	2,07E-02	1,47E-02					
142	*PRDX1*	neg	7,02E-03	1,16E-02					
144	*SWI5*	neg	3,35E-02	3,00E-03					
147	*BTK*	neg	3,92E-02	7,97E-03					

positive (pos), negative (neg). Genes listed by fold change.

**Fig 7:**
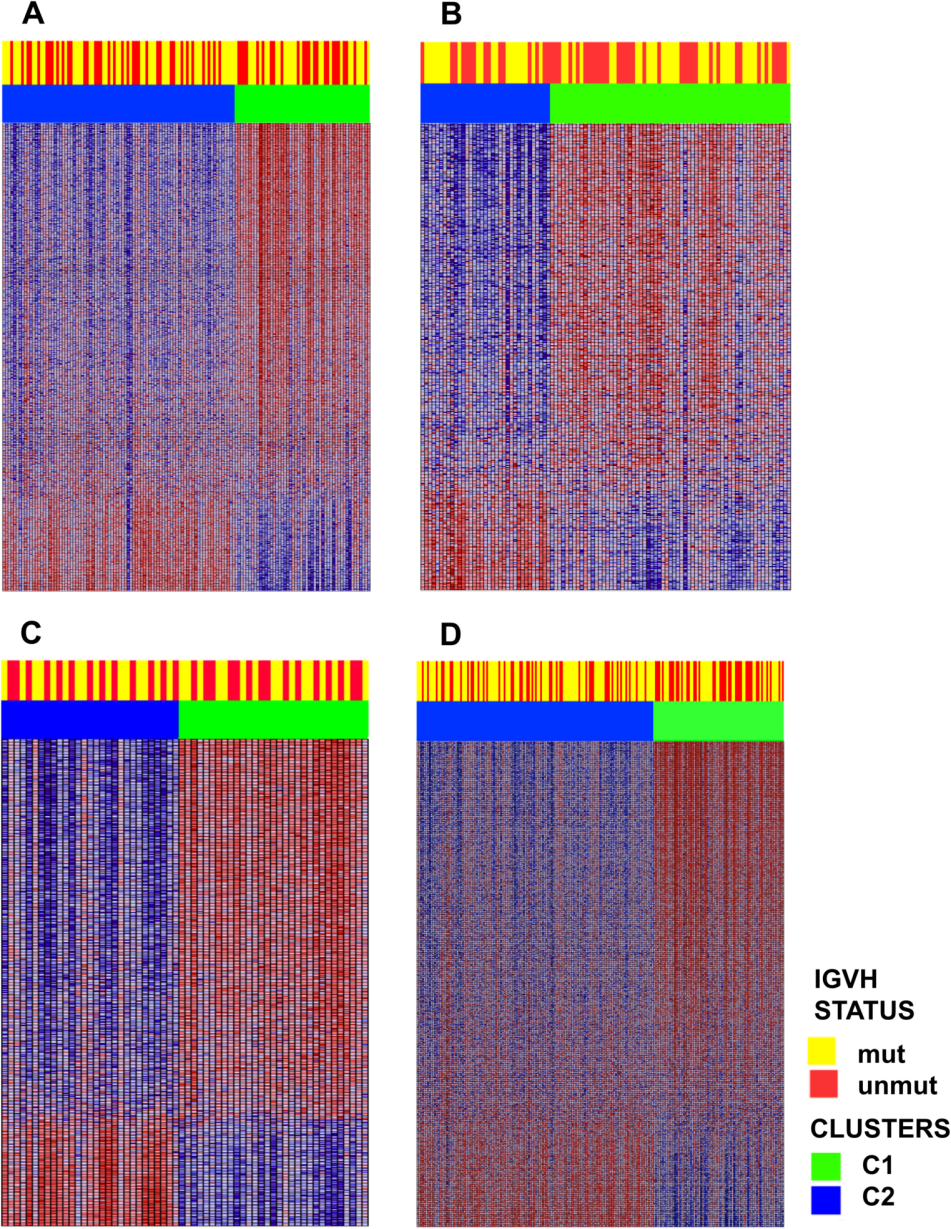
Heatmaps depicting the differential gene expression pattern in clusters cluster 1 and cluster 2 as well as the IGVH status. A, B, C, and D: the GSE38611, GSE2466, GSE9992 and GSE46261 cohorts, respectively. Cluster membership was independent of IGVH mutational status.

Analyzing the clusters and the relationship to IGVH mutational status in the 4 independent cohorts, we found that the segregation of the mutational status was independent of the cluster membership; this was confirmed in all 4 independent cohorts, as seen in the heatmaps of Fig 7. Furthermore, when examining known genes related to IGVH mutational status (genes previously reported in the literature as expressed with a particular pattern in mutated vs. non-mutated IGVH, e.g., *LPL*, *ZAP70*, *CRY1*, and *ZBTB20*), it was found that these markers were not differentially expressed in the clusters.

In conclusion, the survival analysis of the two previously recognized clusters revealed a survival difference that may be attributable to gene expression. Several genes emerged as prognostic markers of survival. The gene expression differences between clusters observed here could provide new information about CLL prognosis that is independent of the IGVH mutational status.

## Discussion

In this paper, using a robust methodology and several cohorts of CLL patients reflecting a broad spectrum of molecular events in the disease, it was possible to distinguish two different patient subgroups and identify subgroup-specific genes. The similarity in the different cohorts, with regard to differential expression patterns between the two identified subgroups, reflects the robustness of the structure. The subdivisions were related with differential clinical outcomes and genes associated with microenvironment and the MAPK and BCR signaling pathways.

The *TCL1A* gene is important in the distinction between clusters due to its up-regulated expression in one of the clusters, reproducibility between cohorts, and its role in the CLL microenvironment and CLL pathogenesis. A high expression level of this proto-oncogene has been associated with causal events in the development of CLL [35, 36, 37]. Sivina *et al* [38] showed that *TCL1A* was among the top genes up-regulated in CLL cells by bone marrow stromal cells (BMSCs). These authors provided evidence that the stromal microenvironment induces *TCL1A* overexpression in CLL cells and represses *TCL1A* target molecules (AP-1 proteins of the FOS/JUN family). Particularly in lymphoid cells, AP-1 proteins can exhibit induction of apoptosis and tumor-suppressive roles [40, 41, 42]. Therefore, these results suggest that *TCL1A* inhibits AP-1-regulated pro-apoptotic activities that normally control B cells.

Interestingly, *TCL1A* and antigen receptors mediated signaling have been previously associated [43, 44, 45]. *TCL1A* seems to acts as a modulator of B-cell receptor–kinase activity, regulating the strength of BCR-mediated cellular activation. The subsequent cellular outcome, associated with apoptosis, growth, inertia, seems primarily determined by a *TCL1A*-dependent (AKT) [44]. The importance of *TCL1A* as a modulator of microenvironment-derived stimuli, suggest its pharmacologic intervention as a treatment rationale for CLL. Therapeutic approaches to disrupt BMSC interactions in CLL are being developed [46, 47], and the present study supports the division of patients based on expression of this gene prior to administration of therapy.

These findings suggest that the microenvironment had a specific influence in patients from cluster 2, this result may be related to the inhibitory activity of critical pro-apoptotic factors that favor cellular survival. Although *TCL1A* showed no statistically significant differences when examined individually (OS: 0,0599; TTT: 0,0626), it is possible that the influence of this gene on patient survival is indirect and is related to its target genes. The *TNFRSF17* gene also support the influence of stromal cells on cluster 2, this gene was the first up-regulated gene identified in experiments in which CLL cells were co-cultured with different stromal cells [38], This gene was identified as one of the most significantly differentially expressed genes between clusters in our merged data.

On the other hand, the *ZNF331* gene is of particular interest for cluster 1 due to its high score in the prediction analysis, this gene is a Kruppel-associated-box zinc-finger protein gene with a role in *TP53* reactivation and induction of tumor cell apoptosis. Nahi et al [48] found evidence of dose-dependent apoptosis and cytotoxicity in CLL cells and suggested that *ZNF331* is a small molecule that targets *TP53*, which could be useful for the treatment of drug-resistant leukemia. In addition, some evidence suggests that *ZNF331* expression in CLL is associated with a higher risk of relapse after treatment, suggesting its use a potential marker for risk [49]. Yu et al [50] recently reported that *ZNF331* is a candidate tumor suppressor gene primarily involved in gastric carcinogenesis, and Vedeld et al [51] found evidence that this gene is methylated in gastrointestinal cancers. Given the role of *ZNF331* as a putative tumor suppressor and the findings demonstrating the important tumor-suppressing functions of zinc-finger proteins and their promising application in cancer therapy, it is worth exploring the functional role of this gene in CLL.

Based on the modular enrichment analysis and the examination of differentially expressed genes, it is possible to speculate that differences in the clusters are due to B-cell receptor (BCR) activation and downstream signaling. The MAPK signaling pathway is one the pathways activated by the BCR receptor [52]. Antigen-dependent BCR activation has been shown to accelerate disease progression in a mouse lymphoma model [53]. Enrichment of the MAPK signaling pathway in CLL is consistent with recent work by Chuang et al [6]; these authors identified gene co-expression subnetworks that were associated with disease progression. In one of these subnetworks, genes in the MAPK signaling pathway had higher expression levels in patients at early stages of the disease.

The groups obtained here are supported by a robust methodology. Different clustering methods have been developed and used to search for structure in gene expression data and extract meaningful biological information. However, each method has limitations, and there is no consensus regarding the best method of clustering. Therefore, we applied different unsupervised methodologies to confirm the structure of the two groups. We applied NMF consensus clustering and hierarchical clustering, and for most of the samples, the class membership results were congruent. NMF clustering appears to have some advantages over other methods, as it is not based on distances and provides a quantitative measure with which to identify the number of clusters. Thus, we used this algorithm for our further analysis to identify cluster markers. NMF clustering has been successfully used in other cancer studies. For example, Collisson et al [54] identified subtypes of pancreatic ductal adenocarcinoma and their differing responses to therapy, and Sadanandam et al [55] proposed a colorectal cancer classification scheme associated with phenotype and responses to therapy.

Without a doubt, unsupervised class discovery in cancer research has led to the identification of subgroups with prognostic implications and generated multiple biomarkers of major importance. However, unsupervised clustering in CLL has been poorly explored, most studies of CLL have been focused on the analysis of known prognostic markers such as IGVH status, cytogenetic aberrations and mutated genes recently identified by next-generation sequencing [1–3, 5]. To our knowledge, the use of unsupervised clustering of expression data in CLL is just beginning to be explored [56]. The present work provides additional information that aids our understanding of this disease, including information about a range of transcriptional markers with potential clinical implications.

## Supporting Information

S1 Fig Consensus matrix, samples and class membership. (PDF)

S1 File Genes that were differentially expressed between clusters based on SAM. Cluster 2 vs. 1 (Table A). Cluster 1 vs. 2 (Table B).
(XLSX)

S2 File Genes with statistically significant differences for survival. Time to treatment (Table A). Overall survival (Table B).
(XLSX)

S1 Table Preprocessed, merged and adjusted data for 237 samples. (TXT)

S2 Table Samples and class membership. (TXT)

S3 Table Genes that were differentially expressed between LN and PB. (TXT)
