# Supplementary figures and images for: Clustering of Expression Data in Chronic Lymphocytic Leukemia Reveals New Molecular Subdivisions
Source: PLoS One. 2015 Sep 10;10(9):e0137132. doi: 10.1371/journal.pone.0137132 (PMC4565688; doi:10.1371/journal.pone.0137132)

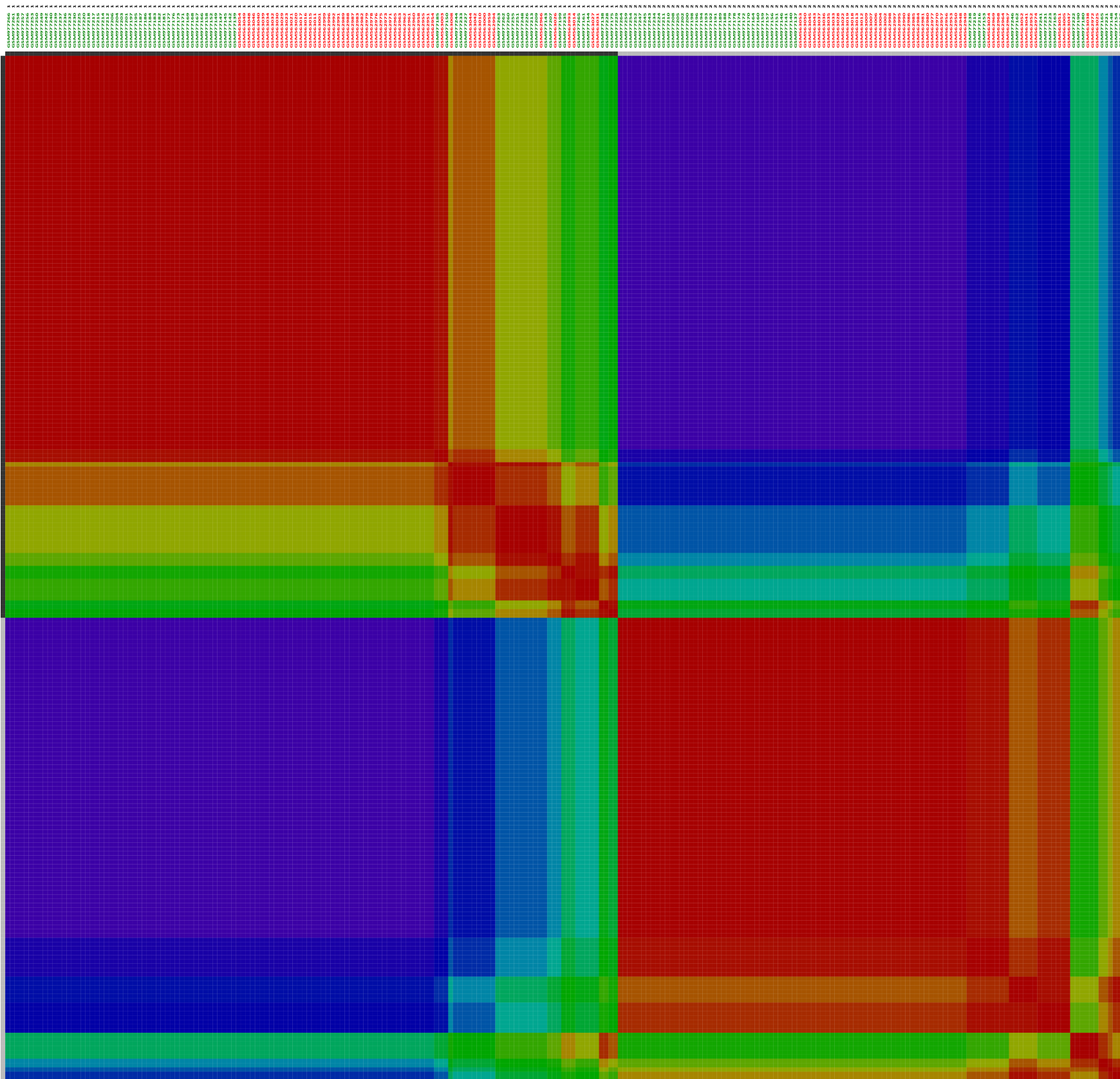

Cohorts:  
GSE39671  
GSE22762

Supplement: S1 Fig — (PDF) [file pone.0137132.s001.pdf]
